# Soziologie der Deglobalisierung

**DOI:** 10.1007/s11609-022-00483-9

**Published:** 2022-09-21

**Authors:** Stefan Schmalz

**Affiliations:** Universität Erfurt, Erfurt, Deutschland

Der Spiegel-Online-Kolumnist Sascha Lobo (2021) bemerkte Ende vergangenen Jahres in einem Essay, dass die Globalisierung sich als „überraschend störanfällig“ erwiesen habe. Preissteigerungen, Lieferengpässe, politische Verwerfungen – all dies habe dazu geführt, dass die Weltwirtschaft labil geworden sei, ja, die Globalisierung sei „irgendwie kaputt“. Mit dieser Sichtweise ist der Autor nicht allein. Die Globalisierung sei im „Rückwärtsgang“, so der Titel eines aktuellen Features im Deutschlandfunk (Becker 2022). Ein Großteil der Öffentlichkeit stehe der Globalisierung überwiegend kritisch gegenüber. Dies ist das Ergebnis einer aktuellen repräsentativen Umfrage im Juli 2022. Heute sehen nur noch 35 % der Deutschen die Globalisierung als Chance. 61 % sehen sie sogar als Risiko. Fünf Jahre zuvor war das Verhältnis fast umgekehrt: Im Juni 2017 bewerteten noch über 60 % die Globalisierung als positiv, weniger als 40 % sahen sie als Risiko (Bidder 2022).

Die Diagnose einer „gescheiterten Globalisierung“ (Flassbeck und Steinhardt 2018), einer „gefesselten Globalisierung“ (Menzel 2021) oder eines „globalization backlash“ (Crouch 2019) hat mittlerweile auch Einzug in der einschlägigen wissenschaftlichen Literatur gehalten. Die globalisierungskritische Konjunktur in den Sozialwissenschaften hat durch die COVID-19-Pandemie und die russische Invasion in der Ukraine einen weiteren Schub bekommen. Denn die Abhängigkeiten und Störpotenziale, die durch die internationale Arbeitsteilung geschaffen wurden, sind heute deutlich sichtbar. Die Krisenphänomene äußern sich in verschiedenen Bereichen wie Lieferengpässen an Halbleitern aus Ostasien oder mangelnder Versorgungssicherheit mit (russischem) Erdgas.

Die Tendenz zur „slowbalisation“, wie die derzeitige Dynamik auf der Titelseite des Economist schon im Januar 2019 bezeichnet wurde, ist dabei nicht neu. Bereits mit der globalen Finanz- und Wirtschaftskrise 2008/09 ist die Globalisierung ins Schlittern geraten. Der Welthandel wächst seitdem nur noch schleppend, der globale Investitionsverkehr ist sogar rückläufig. Flagschiffprojekte wie das transatlantische Freihandels- und Investitionsabkommen TTIP zwischen der EU und den USA sind gescheitert. Zur gleichen Zeit prägen neue politische Konflikte wie der US-amerikanisch-chinesische Wirtschaftskrieg die internationalen Beziehungen. Das Brexit-Votum und die Wahl der Regierung Trump im Jahr 2016 veranschaulichen zudem die Mobilisierungsfähigkeit von rechten globalisierungsfeindlichen Parteien und Bewegungen. Dennoch gibt es auch Prozesse, die auf eine Vertiefung der Globalisierung hindeuten. Im technologischen Bereich weist die Digitalisierung eine große Schwungkraft zur grenzüberschreitenden Vernetzung auf; auch haben internationale Migrationsbewegungen in den letzten zwei Jahrzehnten deutlich zugenommen. Folglich gibt es viele Stimmen in den Sozialwissenschaften, die der These einer „Deglobalisierung“ (Bello 2002) skeptisch gegenüberstehen (Nederveen Pieterse 2021, S. 177 ff.; Scherrer 2022) oder diese schlicht nicht für wünschenswert halten und „nicht weniger, sondern eine veränderte Globalisierung“ anstreben (Baur und Flach 2021). Kurzum: Zur Tiefe und Tragweite der derzeitigen Krise der Globalisierung gibt es unterschiedliche Auffassungen.

## Die Lebenslügen der Globalisierung

Doch worin besteht diese Krise eigentlich?^999^ Der britische Wirtschaftsgeograph David Harvey (1989, S. 240 ff.) hat Globalisierung in einer grundständigen Definition als „Raum-Zeit-Kompression“ bezeichnet. Technologischer Fortschritt und wirtschaftliche Liberalisierung verdichten demzufolge Raum und Zeit, sie lassen räumliche Distanzen schrumpfen und gehen mit einer Intensivierung grenzüberschreitender sozialer Beziehungen einher (s. a. Giddens 1990, S. 64). Technologische Innovation ist demnach ein Globalisierungstreiber. Im Verlauf der Geschichte hat sich die Geschwindigkeit und Effizienz von Transport und Kommunikation gesteigert: vom Segelschiff über den Dampfer bis zum Passagierflugzeug, von der Postkutsche über das Telegramm bis zur WhatsApp-Nachricht. Große Distanzen werden immer schneller überwunden. Um dieses technologische Potenzial zu entfalten, waren politische Initiativen und wirtschaftliche (De‑)Regulierung notwendig. Das British Empire öffnete im 19. Jahrhundert mit seiner Freihandelspolitik und seinen Kanonenbooten den Weltmarkt. Die USA übernahmen später eine vergleichbare Rolle, insbesondere seit der neoliberalen Wende durch die Reagan-Regierung ab den späten 1970er-Jahren. Der Globalisierungsprozess ging dabei in einer Art Bottom-up-Dynamik mit der Entstehung transnationaler Räume und Netzwerke einher, etwa wenn europäische Migrant*innen im 19. Jahrhundert große Strecken auf dem Weg in die „Neue Welt“ Amerika hinter sich brachten, aber weiterhin enge Beziehungen zu ihren Herkunftsorten aufrechterhielten.

Zugleich lief die Globalisierung niemals geradlinig und evolutionär ab, sondern in Schüben (Chase-Dunn et al. 2000; Osterhammel und Petersson 2007): Es kam zeitweise zu schweren Rückschlägen und tiefen Crashs, wie in der Phase der Großen Depression (1929–1939) und des Zweiten Weltkriegs (1939–1945), als die liberale Weltwirtschaftsordnung zerfiel. Nach den dramatischen Krisenerfahrungen orientierten sich viele Staaten um. So versuchten etwa verschiedene lateinamerikanische Länder, sich seit den 1930er-Jahren ein Stück weit vom Weltmarkt abzukoppeln und mit der Hilfe von Importsubstitution eigene Industriestrukturen aufzubauen (Halperín Donghi 1994, S. 411 ff.). Solche Phasen der Deglobalisierung, die sich durch einen Verfall globaler Institutionen und einen Rückbau transnationaler Verflechtungen und Netzwerke auszeichneten, wurden jedoch schließlich überwunden, ja, mündeten später sogar in neuen Globalisierungswellen. Nach der Großen Depression erreichte der Welthandel zwar erst in den frühen 1970er-Jahren ein ähnliches Verflechtungsniveau wie vor dem Ersten Weltkrieg (Steger 2017, S. 33); doch bereits am Ende des Jahrzehnts startete die aktuelle Globalisierungsphase, die die Verflechtung der Weltwirtschaft auf neue Höhen trieb.

Die aktuelle Globalisierungsphase wurde durch verschiedene Faktoren begünstigt: den Durchbruch neuer digitaler Technologien, das Ende des Ostblocks, die Außenöffnung Chinas und die Gründung neuer Institutionen der Global Governance wie der Welthandelsorganisation im Jahr 1994. Nicht nur wirtschaftliche Indikatoren wie Welthandel, Direktinvestitionen oder Finanzmarktransaktionen haben sich vervielfacht (Robinson 2004), sondern auch soziale Interaktionen in Kommunikation (Telefon- und Videoanrufe, soziale Netzwerke, World Wide Web) oder Kultur (Massentourismus, Studierendenaustausch) haben sich vertieft (Deutschmann 2021). Globalisierung und Transnationalisierung haben auf diese Weise auch räumliche und staatliche Ordnungen verändert (Löw et al. 2021; Zürn 1998): Diese Dynamiken wurden teils als so tiefgreifend begriffen, dass sie vielen Beobachter*innen als historisches Novum und sogar als unumkehrbar galten (Beck 1997, S. 17 ff.; Robinson 2004). Wie bereits in früheren Globalisierungsperioden lassen sich jedoch heute Risse im transnationalen Gefüge beobachten. Diese äußern sich entlang dreier zentraler Bruchlinien: a) zwischen Staaten, b) innerhalb von Gesellschaften und c) bei deren jeweiliger sozialökologischer Einbettung. Diese Risse offenbaren die Lebenslügen des Globalisierungsprojekts, das in den späten 1970er-Jahren begonnen hatte. Zunächst gingen viele Autor*innen davon aus, dass Globalisierung zu einer allgemeinen Einkommenskonvergenz beitragen würde und sich Länder wie China oder Indien als verantwortungsvolle Stakeholder in die liberale Weltwirtschaftsordnung integrieren würden (Wolf 2005; Ikenberry 2008). Heute ist jedoch recht offenkundig, dass die Globalisierung nicht nur ungleich verlaufen ist, sondern auch zu zwischenstaatlichen Konflikten beigetragen hat. Der Hegemonialkonflikt des 21. Jahrhunderts zwischen China und den USA dreht sich um den Modus der Globalisierung und die Machtverteilung im internationalen System (Mearsheimer 2021; Hung 2022; Schmalz 2018). Der chinesische Parteistaat verfolgt ein staatsgetriebenes Entwicklungsmodell und treibt seine Internationalisierung mit der Hilfe von Fünf-Jahresplänen, Subventionen und Staatsunternehmen voran. Die USA haben im Gegenzug der Volksrepublik keine Partnerschaft angeboten, sondern eine Containment-Strategie entwickelt. Der zunächst latente Konflikt hat sich seit der Regierung Trump zu einem offenen Wirtschaftskrieg mit Importzöllen, Investitionskontrollen und Sanktionen gegenüber chinesischen (Digital‑)Unternehmen gewandelt (Schmalz et al. 2022). Die russische Invasion in der Ukraine im Februar 2022 verschärft den Eindruck einer „Verwilderung“ internationaler Beziehungen. Bereits jetzt haben solche internationale Konflikte Auswirkungen auf transnationale Netzwerke: Die globale „Netzwerkgesellschaft“ (Castells 2001) sieht sich in China und Russland neuen Barrieren gegenüber (Pohle und Voelsen 2022), Russland ist vom westlichen Finanzsystem weitgehend abgekoppelt (Kagarlitsky et al. 2022), neue Restriktionen erschweren die Einreise chinesischer Student*innen in die USA^1^, das „geopolitische Risiko“ (Suder und Kallmorgen 2022) beeinträchtigt globale Wertschöpfungsketten.

Die Globalisierung wird jedoch nicht nur *zwischen*, sondern auch vermehrt von politischen Kräften *innerhalb* der traditionellen Industriestaaten zur Disposition gestellt. Anders als von vielen Beobachter*innen propagiert (Bourguignon 2013; Beck und Sznaider 2006), hat die jüngste Globalisierungswelle nicht nur Wohlstandsgewinne mit sich gebracht und zu Kosmopolitismus und kultureller Hybridisierung beigetragen, sondern vielmehr auch zu Fragmentierung und Deprivationserfahrungen geführt – Marktöffnungen gingen bisweilen mit Produktionsverlagerung und Deindustrialisierung einher, während Migration und kultureller Wandel mancherorts zu Ressentiments und Abwehrhaltungen beitrugen. Diese Dynamik war wiederum eine Triebfeder für Deglobalisierung, wie sich an der Wahl der Regierung Trump (2017–2021) darstellen lässt: Der republikanische Kandidat Trump warb im US-Wahlkampf 2016 erfolgreich um die Stimmen der Arbeiterschaft im Rust Belt, die besonders unter der Deindustrialisierung gelitten hatte und für die Rhetorik eines „Make America Great Again“ sowie protektionistische Tonlagen empfänglich war (Schmalz 2021). Ähnliche rechtspopulistische Formationen und autoritäre „Bewegungen polanyischen Typs“ (Dörre 2019b, S. 226), die auf eine rechte Globalisierungskritik setzen und die Folgen „ungezügelter Migration“ und „unfairen Standortwettbewerbs“ problematisieren, sind in Europa wirkmächtig geworden – die Brexit-Kampagne von 2016 ist das wohl bekannteste Beispiel.^2^ In Deutschland kultiviert die AfD eine Erzählung, wonach eine übermächtige transnationale „bunte“ Klasse, die in „international agierenden Unternehmen, in Organisationen wie der UN, in den Medien, Start-ups, Universitäten, NGOs, Stiftungen, in den Parteien und ihren Apparate[n]“ (Gauland 2018) präsent ist, einen zersetzenden Einfluss auf Heimat und Nation ausübe. Dieser Backlash treibt protektionistische Politik voran (Walter 2021), gerade wenn etablierte Parteien Teile des globalisierungskritischen Diskurses übernehmen.

Nicht zuletzt war mit der Globalisierung ein weitgehender Technik- und Gestaltungsoptimismus verbunden. Mit der Steigerung von Mobilität und Warenverkehr wurde jedoch auch der Naturverbrauch erhöht: Ungebremste Globalisierungsfantasien externalisierten lange Zeit schon im Ansatz die immensen ökologischen Kosten einer wachsenden Weltwirtschaft (Lessenich 2016), allen frühen Warnungen vor den „Grenzen der Globalisierung“ (Altvater und Mahnkopf 1996) zum Trotz. Durch die Klimakrise sind die Kosten dieser globalen sozioökonomischen Beschleunigung indes manifest geworden. Die politische Zielsetzung von Klimaneutralität erzeugt sozialökologischen Transformationsdruck, und die Auswirkungen des Klimawandels erfordern eine erhöhte Resilienz von Wertschöpfungs- und Logistiknetzwerken. So verursacht allein der globale Warenhandel rund 30 % der CO_2_-Emmissionen des Transportsektors und etwa 7 % der Gesamtemissionen. Der Schiffverkehr würde durch alternative Kraftstoffe wie Methanol oder Ammoniak deutlich höhere Betriebskosten nach sich ziehen, beim Flugverkehr können fossile Energieträger aufgrund des hohen Stromverbrauchs nicht unmittelbar durch E‑Fuels ersetzt werden (Cames et al. 2021; Stolz et al. 2022). Die Folge eines sozialökologischen „reembedding“ des Welthandels wären steigende Kosten und eine Entschleunigung des Güterhandels.^3^ Hinzu kommen nicht zuletzt die *Auswirkungen* des Klimawandels. Denn dieser bedroht nicht nur ökologische Grundlagen, sondern auch den reibungslosen globalen Warenverkehr: Flutkatastrophen, wie etwa in der chinesischen Provinz Henan im vergangenen Jahr, oder anhaltende Hitzewellen wie im Sommer 2022 haben zu großen Schäden geführt und Produktionsstandorte in globalen Lieferketten weitgehend lahmgelegt. Folglich ist eine Relativierung der Kostenvorteile durch globale Warenketten zu erwarten.

Die skizzierten Bruchlinien wirken ineinander: Geopolitische Konflikte bremsen die sozialökologische Transformation aus, nationalistische Bewegungen senken die Bereitschaft zur internationalen Kooperation und rechte Parteien leugnen oder relativieren wiederum den Klimawandel. Eine mögliche – wenn nicht die wahrscheinliche – Folge davon ist eine sich selbst verstärkende Konstellation, die die Globalisierung bremst oder sogar deren Rückbau impliziert.

## Zeithorizonte der (De‑)Globalisierung: 50, 250, 500 Jahre

Bei der Bewertung der Tragweite und Dynamik dieses Wandels gibt es unterschiedliche Sichtweisen. Die Bestimmung des Charakters der derzeitigen Globalisierungsphase steht dabei in engem Zusammenhang mit der Definition ihres Ausgangspunkts. Denn Globalisierung und Deglobalisierung können aus unterschiedlichen zeitlichen Perspektiven begriffen werden. In der sozialwissenschaftlichen Diskussion sind drei unterschiedliche Lesarten gängig: Eine weite Interpretation sieht Globalisierung als einen Prozess mit einer *longue durée*, der bereits mit dem europäischen Kolonialismus begann, sich später mit dem British Empire vertiefte und auf diese Weise ein weltumspannendes System schuf (Wallerstein 2000, S. 71 ff., 221 ff.). Diese 500-Jahre-Perspektive sieht zentrale Merkmale dieses Weltsystems bereits im langen 16. Jahrhundert entstehen, darunter v. a. globale räumliche Ungleichheiten, Kapitalakkumulation als Organisationsprinzip der Wirtschaft, Eurozentrismus und Rassismus (Boatcă 2015; Quijano 2000). Viele heutige Strukturmerkmale der Weltwirtschaft haben aus dieser Perspektive koloniale Vorläufer, so zum Beispiel in der Arbeitsteilung beim europäischen Schiffbau des 17. Jahrhunderts, der stark von internationalen Ressourceninputs (Holz, Flachs, Teer) abhing (Hopkins und Wallerstein 1986). Deglobalisierung wird aus dieser Sichtweise vereinzelt als eine Form des katastrophischen Zusammenbruchs des gesamten Weltsystems (Wallerstein 2013), aber gemeinhin als ein zyklischer Trend beschrieben (Chase-Dunn et al. 2000; Arrighi und Silver 1999). Hegemonialmächte lösten einander demzufolge historisch ab und trieben die Verflechtung der Weltwirtschaft in neuen Phasen der globalen Hegemonie immer weiter voran. Globalisierung wird folglich in nicht-hegemonialen Phasen immer wieder von Deglobalisierungsperioden unterbrochen. Sie waren ein Anzeichen von systemischem Chaos, das von Krieg und Krise gezeichnet ist. Die derzeit anbrechende Epoche lässt sich daher aus dieser historischen Makro-Perspektive bereits als eine antizipieren, die durch einen Niedergang der Weltwirtschaft charakterisiert sein wird.

Eine zweite Lesart sieht den Aufstieg des Industriekapitalismus im 18. Jahrhundert als eigentlichen Anfangspunkt der Globalisierung. Die Entstehung des Bürgertums, der Dampfschifffahrt und der mechanisierten Industrie war demnach gleichbedeutend mit dem Beginn der Steigerungslogik und des exponentiellen Wirtschaftswachstums der kapitalistischen Ära. Das British Empire garantierte zollfreien Handel in großen Teilen der Welt, und unter britischer Vorherrschaft wurde, wie Marx beschrieb, „die Produktion und Konsumtion aller Länder kosmopolitisch gestaltet“ (Marx und Engels 1972 [1848], S. 466). Die gewaltigen Produktivitätsfortschritte und militärischen Innovationen machten es demnach möglich, dass Europa und die USA Asien als das Zentrum der Weltwirtschaft ersetzten. Die USA übernahmen später die Rolle Großbritanniens als Hegemon, trieben den Fordismus weltweit voran und prägten die aktuelle, finanzmarktgetriebene Globalisierungsphase, die aus der 250-Jahre-Perspektive eine neue Kapitalismusformation darstellt (Cox 1987). Deglobalisierungsprozesse sind aus dieser Sicht ein Produkt von schockartigen Krisen, Kapitalvernichtung und kriegerischen Auseinandersetzungen: Das Zeitalter des Imperialismus und der Erste Weltkrieg ergaben sich somit zwar aus den Widersprüchen von Weltwirtschaft und -politik, waren aber nur schwer vorhersehbar. In der derzeitigen Phase der „Deglobalisierung 2.0“ (van Bergeijk 2019) erkennt diese 250-Jahre-Perspektive durchaus Parallelen zum Verfall der britischen Freihandelsordnung, betont aber zugleich die Kontingenz eines solchen Prozesses. Eine größere Tragweite wird dem fossilen Energieregime (Kohle, Erdöl) zugemessen: Mit der ökologischen Krise gerät demnach auch die Steigerungslogik des fossilen Kapitalismus, ja, der kapitalistischen Moderne an die Grenzen, was Degrowth und Deglobalisierung vorantreiben könnte (Rosa et al. 2017; Gudynas 2019).

Die dritte Interpretationslinie ist heute vorherrschend im wissenschaftlichen Diskurs. Sie betont die historisch neue Qualität des Globalisierungsprozesses seit den späten 1970er-Jahren und lässt sich als 50-Jahre-Perspektive bezeichnen. Sie hebt dabei insbesondere auf die Transnationalisierung von sozialen Räumen, Kultur, Wirtschaft, Kommunikation, Politik, Sozialstruktur und Migration ab (Pries 2002). Technologische Treiber der Transnationalisierung sind ihr zufolge die digitalen Informationstechnologien (Castells 2001), wirtschaftliche Push-Faktoren sind die „neue internationale Arbeitsteilung“ (Fröbel et al. 1977) mit dem Aufstieg transnationaler Konzerne und Produktionsnetzwerke sowie der globale „Finanzmarktkapitalismus“ (Windolf 2005). Transnationalisierung gehe dabei auch mit Umbrüchen in der Sozialstruktur einher wie der Herausbildung transnationaler Klassen, Ungleichheiten und Migrationsnetzwerke (Sklair 2001; Weiß 2005; Faist und Bilecen 2020). Auf der politischen Ebene haben Institutionen der Global Governance (Zürn 1998) und die Aufwertung subnationaler Räume wie Global Cities (Sassen 1991) die Rolle des Nationalstaats relativiert; Staatlichkeit überschreitet heute den nationalen Rahmen (Beck 1997, S. 55 ff., 183 ff.; Brand et al. 2007). Eine zentrale Implikation dieses 50-Jahre-Ansatzes besteht – paradoxerweise ähnlich zur 500-Jahre-Perspektive – darin, den „methodologischen Nationalismus“ (Wimmer und Glick Schiller 2002) und damit den Untersuchungsgegenstand und die Analyseeinheiten sozialwissenschaftlicher Forschung zu überdenken (Pries 2005). So stehen transnationale Räume und Netzwerke wie transnationale Familien und Biografien oder globale Wertschöpfungsketten im Mittelpunkt der Forschung. Deglobalisierung impliziert aus dieser Perspektive darum auch einen Rückbau transnationaler Räume und Interaktionen, was wiederum auf Skepsis stößt. Denn im Falle globaler Wertschöpfungsketten wird unter dem Schlagwort „Reshoring“ zwar rege über Rückbauprozesse diskutiert (Gong et al. 2022), beobachtbar sind aber bisher allenfalls ein Stocken der Internationalisierung oder eine Verschiebung transnationaler Produktionsnetzwerke (Butollo und Staritz 2022). Aktuelle Zeitdiagnosen zur Krise der Globalisierungsperiode bezeugen deren anhaltende gesellschaftliche Auswirkungen und betonen etwa, dass sich die kapitalistische „Hyperglobalisierung“ nationalstaatlich-demokratischer Kontrolle und Regulierung entziehe und zu neuen sozialen Konflikten führe (Rodrik 2011; Streeck 2021).

Die unterschiedlichen Zeithorizonte^4^ der Analysen bringen also unterschiedliche soziologische Zeitdiagnosen hervor. Das Changieren zwischen Bejahung und Ablehnung der Deglobalisierungsthese, zwischen ihrer Interpretation als zyklisches Muster oder historische Neuheit sowie zwischen den unterschiedlichen Bewertungen der Bruchlinien der Globalisierung führt zu disparaten Antworten auf die Frage, worin die derzeitige Krise der Globalisierung besteht und wie ihre Tragweite einzuordnen ist.

## Deglobalisierung: Ein neues Forschungsfeld

Die Analyse der aktuellen Deglobalisierungsdynamiken ist demnach ein neues Forschungsfeld, das an die klassischen Studien zur Globalisierung und Transnationalisierung anknüpft. Die zentralen Fragen sind dabei nicht nur, „ob“ und „wie“ Deglobalisierung stattfindet, sondern auch, „wer“ oder „was“ sie vorantreibt. Zur Bewertung, ob überhaupt Deglobalisierung erfolgt, ist es hilfreich, an klassische Globalisierungsdefinitionen zu erinnern, die zwischen verschiedenen Dimensionen von Globalisierung unterscheiden (Held et al. 1999; Steger 2017). Denn bei der wirtschaftlichen, politischen, technologischen und kulturellen Globalisierung lassen sich, wie auch im Falle der Deglobalisierung, Ungleichzeitigkeiten erkennen. Während sich in Teilen der Wirtschaft und auch in der Politik – wie z. B. beim Ausschluss Russlands aus dem globalen Finanzsystem und der G8 –Tendenzen einer Deglobalisierung beobachten lassen, bleiben derartige Entwicklungen in Kultur und Technologie weitaus schwächer ausgeprägt. Je nach Untersuchungsgegenstand kommen Analysen deshalb zu divergierenden Schlussfolgerungen, ohne jedoch die Wechselwirkungen zwischen den einzelnen Dimensionen genauer zu beleuchten.

Dieses Problem berührt auch die Frage nach den zentralen Treibern der Krise der Globalisierung. Bisher scheint die gegenwärtige Tendenz zur Deglobalisierung eine – durchaus chaotische und nur begrenzt vorhersehbare – Top-Down-Bewegung zu sein, die vor allem von politischen Akteuren ausgeht: Regierungen agieren und reregulieren, während andere gesellschaftliche Akteure, darunter auch Wirtschaftsakteure, sich primär dazu verhalten und reagieren (de Graaff und Valeeva 2021, S. 1168 f.). Die Transnationalisierung der sozialen Welt scheint die Deglobalisierung in vielen Bereichen eher auszubremsen. Eine eigenständige Deglobalisierung „from below“ findet bisher nur in Ausnahmefällen statt. Darunter fällt beispielsweise der freiwillige Rückzug vieler transnationaler Unternehmen vom russischen Markt nach dem Beginn des Ukraine-Konflikts (Kagarlitsky et al. 2022) oder etwa der Verzicht auf Fernreisen bestimmter gesellschaftlichen Milieus aus ökologischen Gründen. Ähnlich wie die Wechselwirkungen zwischen einzelnen Dimensionen von Globalisierung und Deglobalisierung erscheint auch die Analyse des Verhältnisses von Makro- und Mikrodynamiken als lohnenswert.

Neben dem Verhalten von Akteuren spielt dabei auch die Frage, auf welche Weise sich Deglobalisierungsprozesse vollziehen, eine wichtige Rolle. In der Debatte um den „Globalisierungs-Backlash“ ist etwa auffällig, dass jeweils einschneidende Ereignisse wie die globale Finanzkrise 2008/09, der Brexit 2016, die Corona-Pandemie ab 2020 oder auch der Ukrainekrieg 2022 sozialwissenschaftliche Diskussionen um Deglobalisierung initiierten. Diese Ereignisse, bei denen die Globalisierung „repulsiv“ (Dörre 2019a, S. 35 ff.) auf die westlichen Demokratien zurückschlägt, können als eine Form des „deglobalization by desaster“ gedeutet werden, die zu notgedrungenem Akteurshandeln führt. Dem Handeln in solchen Ausnahmesituationen steht bisher nur vereinzelt ein selektives, strategisch orientiertes „deglobalization by design“, wie durch die chinesische duale Zirkulationsstrategie oder durch neuere defensive EU-Gesetzgebungen in der Außenwirtschaft, gegenüber (Schmalz et al. 2022). Das Zusammenwirken von – teilweise katastrophischen – Ereignissen und bewussten langfristigen Strategien scheint bei der Bewertung der aktuellen Entwicklungsdynamik wesentlich zu sein.

Aus einer soziologischen Perspektive gilt es, diese Fragen und Themenkomplexe zu bearbeiten. Das vorliegende Schwerpunktheft soll hierzu einen Beitrag leisten. In seinem einleitenden Artikel schreibt Ulrich Menzel (1998) sein Werk *Globalisierung versus Fragmentierung* fort. Menzel vertritt die These eines Epochenbruchs: Die „entfesselte“ Globalisierung der 1990er-Jahre sei einer „gefesselten“ Globalisierung gewichen. Dies sei kein historisches Novum. Als ein wellenförmiger Prozess werde Globalisierung stets von Prozessen der Fragmentierung begleitet, die Erstere ausbremsen, ja sogar zurückwerfen können. Menzel sieht dabei zwei notwendige Bedingungen für eine fortschreitende Globalisierung: Große Mächte, die globale öffentliche Güter bereitstellen, und einen hegemonialen Rechtfertigungsdiskurs, der die Liberalisierung der Weltwirtschaft legitimiert. Heute sind seiner Einschätzung nach mit dem „US-Decline“ und dem Aufstieg Chinas sowie der Krise neoliberaler Denkmuster die Säulen der aktuellen Globalisierungsperiode ins Wanken geraten. Menzel benennt verschiedene Kippunkte wie die Lehman-Pleite 2008 und die COVID-19-Pandemie seit 2020 als Zäsuren, die zum Stocken der Globalisierung beigetragen haben.

Der Beitrag von Florian Butollo und Cornelia Staritz interveniert in die aktuelle Diskussion um die geografische Restrukturierung globaler Produktionsnetzwerke. Aus einer Global-Value-Chain-Perspektive untersuchen die Autor*innen die Auswirkungen der COVID-19-Pandemie auf die globale Fertigung. In ihrer Studie zur Automobil‑, Bekleidungs- und Elektronikindustrie melden sie dabei Zweifel an der These einer umfassenden Deglobalisierung an. Reshoring, Backshoring oder Nearshoring sind, so der Befund der Autor*innen, nur *eine* von verschiedenen Unternehmensstrategien. Ungeachtet neuer Störfaktoren wie Handelskriege, steigender Lohnkosten oder auch umweltpolitischer Reaktionen auf die ökologische Krise weise der Trend bisher eher in Richtung einer Diversifizierung globaler Produktionsstrukturen. Allerdings könnten anhaltende Störungen langfristig zu einer verstärkten Regionalisierung und Lokalisierung globaler Wertschöpfung führen.

Stefan Schmalz, Helena Gräf, Philipp Köncke und Lea Schneidemesser untersuchen die Reaktionen der USA und der EU auf den Aufstieg chinesischer High-Tech-Unternehmen. Aus Sicht der Vergleichenden Politischen Ökonomie beschreiben sie die chinesische Wirtschaft als eine Form des hybriden Parteistaatskapitalismus, bei dem politische und wirtschaftliche Macht eng verschränkt sind. Die staatsgetriebene Globalisierung Chinas trägt darum den Autor*innen zufolge nicht nur zu wirtschaftlicher Konkurrenz, sondern auch zu Sicherheitsbedenken und politischen Reaktionen in vielen westlichen Ländern bei. Anhand der Felder Außenhandel, Investitionen, Hochtechnologie und Industriepolitik zeigen die Autor*innen, dass die USA auf eine aggressive Handels- und Sanktionspolitik gegenüber China setzen, während die EU überwiegend mit defensiveren Maßnahmen reagiert. Eine stärkere Fragmentierung der Weltwirtschaft ist somit ein reales Entwicklungsszenario.

Aus einer netzwerktheoretischen Perspektive analysieren Julia Pohle und Daniel Voelsen den Strukturwandel der globalen digitalen Ordnung. Sie beobachten, dass Staaten und Unternehmen sich darum bemühen, ihre Souveränität und Macht über Teilnetze des World Wide Web zu festigen. Dabei untersuchen sie die Globalisierung von Internetinfrastruktur und Internetanwendungen und deren politische Regulierung und schlagen eine zeithistorische Phaseneinteilung der Netzwerkstruktur des Internets vor: Auf eine Pionierphase eines offenen Netzwerkes unter Führung der USA bis in die 1990er-Jahre folgen eine globale Ausweitung und Pluralisierung des Internets in den 2000er-Jahren und zuletzt eine wachsende Dezentralisierung und machtpolitische Regulierung der Netzwerkstruktur in China oder Russland, aber auch in Demokratien und privatwirtschaftlich monopolisierten digitalen Bereichen. Die Folge dieser Entwicklung sei zwar keine Fragmentierung des Internets, absehbar aber sei eine Rekonfiguration mit stärker regulierten Teilnetzen.

Der russische Soziologe Boris Kagarlitsky beschreibt in einem Gespräch mit Janina Puder und Stefan Schmalz die Folgen der russischen Invasion in der Ukraine für die globale politische Ökonomie. Er analysiert die Auswirkungen der westlichen Sanktionen auf den russischen Finanzsektor und die Industrie und äußert sich kritisch zu den Entwicklungsperspektiven der russischen Wirtschaft. Den russischen Angriffskrieg sieht er als Teil einer globalen Strukturkrise, für den die Widersprüche und Legitimationsdefizite oligarchischer Herrschaft eine wichtige Triebkraft sind. Kagarlitsky diskutiert die globalen Auswirkungen des Konflikts und prognostiziert eine Phase der Deglobalisierung. Bei seiner Einschätzung bezieht er sich auf soziologische Klassiker wie Wallerstein und Weber sowie die russische sozialwissenschaftliche Diskussion.

## Footnotes

999 Einige Argumente hierzu habe ich bereits in Form eines Essays formuliert, siehe Schmalz 2020.

1 Solche Barrieren existieren auch bei der Einreise nach China, die seit Beginn der Pandemie für ausländische Studierende kaum möglich ist. Über ein Drittel aller Ausländer*innen haben China seitdem verlassen. Es bleibt eine offene Frage, wie sich die Einreisebestimmungen in den kommenden Jahren vor dem Hintergrund der Zero-COVID-Politik entwickeln werden.

2 Lange Zeit war die Kritik an der Globalisierung ein Alleinstellungsmerkmal der politischen Linken – Organisationen wie Attac dominierten die globalisierungskritische Debatte mit Themen wie der Finanztransaktionssteuer (Altvater und Mahnkopf 1996; Sassen 1999; Leggewie 2003). Die politische Rechte, die in der globalisierungskritischen Diskussion eine eher randständige Position besetzte (Leggewie 2003, S. 50 ff.), hat das Thema in der öffentlichen Diskussion neu besetzt (Bello 2021). Dies ist jedoch kein Automatismus; die auffälligen Überschneidungen bei dem Wählerklientel von Donald Trump und Bernie Sanders zeigen, dass Deglobalisierungsimpulse auch von der politischen Linken ausgehen können (Schirm 2019).

3 Zu diesen Mehrausgaben kommen weitere Kosten für die Herstellung von „schmutzigen“, energieintensiven Produkten ohne ökologische Auflagen: So werden mit dem geplanten EU-weiten Carbon Border Adjustment Mechanism (CBAM) de facto Importzölle für Produkte wie Aluminium, Stahl und Zement verhängt (Erixon et al. 2022).

4 An dieser Stelle sei noch erwähnt, dass einige Studien über den 500-Jahre-Zeitraum hinausgehen. So zum Beispiel Göran Therborns (2000, S. 159 ff.) Analyse, die sechs historische Globalisierungswellen ausmacht, darunter etwa die Verbreitung und Expansion der Weltreligionen vom 4. bis zum 7. Jahrhundert. Eine weitere bekannte Arbeit ist Janet Abu-Lughods (1991) Werk zur gescheiterten Herausbildung eines asiatisch dominierten Weltsystems im 13. und 14. Jahrhundert.

## References

[CR1] Abu-Lughod JL (1991). Before European hegemony: The world system A.D. 1250–1350.

[CR2] Altvater E, Mahnkopf B (1996). Grenzen der Globalisierung. Ökonomie, Ökologie und Politik in der Weltgesellschaft.

[CR3] Arrighi G, Silver BJ (1999). Chaos and governance in the modern world system.

[CR4] Baur, A., & Flach, L. (2021). Nicht weniger, sondern eine veränderte Globalisierung. *Makronom.de *vom 16.09.2021. https://makronom.de/lieferketten-welthandel-nicht-weniger-sondern-eine-veraenderte-globalisierung-40203. Zugegriffen: Aug. 2022.

[CR5] Beck U (1997). Was ist Globalisierung? Irrtümer des Globalismus – Antworten auf Globalisierung.

[CR6] Beck U, Sznaider N (2006). Unpacking cosmopolitanism for the social sciences: a research agenda. The British Journal of Sociology.

[CR7] Becker, M. (2022). Globalisierung im Rückwärtsgang. Warum Unternehmen nach Deutschland zurückkehren. *Deutschlandfunkkultur.de *vom 01.08.2022. https://www.deutschlandfunkkultur.de/globalisierung-im-rueckwaertsgang-100.html. Zugegriffen: Aug. 2022.

[CR9] Bello W (2002). Deglobalization. Ideas for a new world economy.

[CR8] Bello W (2021). Deglobalisierung – Zwanzig Jahre später: (Zur Diskussion). Peripherie.

[CR10] van Bergeijk PAG (2019). Deglobalization 2.0. Trade and openness during the great depression and the great recession.

[CR11] Bidder, B. (2022). Die Deutschen wenden sich von der Globalisierung ab. *Spiegel Online *vom 01.08.2022. https://www.spiegel.de/wirtschaft/deutschland-die-mitte-verliert-den-glauben-an-die-globalisierung-a-6e80a351-4769-438e-bd54-f590aef4b917. Zugegriffen: Aug. 2022.

[CR12] Boatcă M (2015). Global inequalities beyond occidentalism.

[CR13] Bourguignon F (2013). Die Globalisierung der Ungleichheit.

[CR14] Brand U, Görg C, Wissen M (2007). Verdichtungen zweiter Ordnung: Die Internationalisierung des Staates aus einer neo-poulantzianischen Perspektive. PROKLA.

[CR15] Butollo F, Staritz C (2022). Deglobalisierung, Rekonfiguration oder Business as Usual? COVID-19 und die Grenzen der Rückverlagerung von globalen Produktionsnetzwerken. Berliner Journal für Soziologie.

[CR16] Cames, M., Chaudry, S., Göckeler, K., Kasten, P., & Kurth, S. (2021). E‑fuels versus DACCS. Total costs of electro-fuels and direct air capture and carbon storage while taking into account direct and upstream emissions and environmental risks. https://www.transportenvironment.org/wp-content/uploads/2021/08/2021_08_TE_study_efuels_DACCS.pdf. Zugegriffen: Aug. 2022.

[CR17] Castells M (2001). Das Informationszeitalter.

[CR18] Chase-Dunn C, Kawano Y, Brewer BD (2000). Trade globalization since 1795: waves of integration in the world-system. American Sociological Review.

[CR19] Cox RW (1987). Production, power, and world order. Social forces in the making of history.

[CR20] Crouch C (2019). The globalization backlash.

[CR21] Deutschmann E (2021). Mapping the transnational world. How we move and communicate across borders, and why it matters.

[CR23] Dörre K, Ketterer H, Becker K (2018). Demokratie statt Kapitalismus oder: Enteignet Zuckerberg!. Was stimmt nicht mit der Demokratie? Eine Debatte mit Klaus Dörre, Nancy Fraser, Stephan Lessenich und Hartmut Rosa.

[CR22] Dörre K (2019). „Take back control!“ Marx, Polanyi, and right-wing populist revolt. Österreichische Zeitschrift für Soziologie.

[CR24] Erixon, F., Lamprecht, P., Montero, R. Z., & Sharma, V. (2022). *The new wave of defensive trade policy measures in the European Union: Design, structure, and trade effects.* ECIPE Occasional Paper, 04/2022. https://ecipe.org/publications/new-wave-of-defensive-trade-policy-measures-in-eu/. Zugegriffen: Aug. 2022.

[CR25] Faist T, Bilecen B, Faist T (2020). Der transnationale Ansatz: Transnationalisierung, Transnationale Soziale Räume, Transnationalität. Soziologie der Migration.

[CR2018] Flassbeck H., & Steinhardt, P. (2018). *Gescheiterte Globalisierung. Ungleichheit, Geld und die Renaissance des Staates*. Berlin: Suhrkamp.

[CR26] Fröbel F, Heinrichs J, Kreye O (1977). Die neue internationale Arbeitsteilung. Strukturelle Arbeitslosigkeit in den Industrieländern und die Industrialisierung der Entwicklungsländer.

[CR27] Gauland, A. (2018). Warum muss es Populismus sein? *Frankfurter Allgemeine Zeitung *vom 06.10.2018. https://www.faz.net/aktuell/politik/inland/alexander-gauland-warum-muss-es-populismus-sein-15823206.html. Zugegriffen: Aug. 2022

[CR1990] Giddens, Anthony (1990). *The consequences of modernity*. Cambridge: Polity.

[CR28] Gong H, Hassink R, Foster C, Hess M, Garretsen H (2022). Globalisation in reverse? Reconfiguring the geographies of value chains and production networks. Cambridge Journal of Regions, Economy, and Society.

[CR29] de Graaff N, Valeeva D (2021). Emerging Sino–European corporate elite networks. Development and Change.

[CR30] Gudynas E, Ramírez M, Schmalz S (2019). Extraktivismen. Erscheinungsformen und Nebenwirkungen. Extraktivismus. Lateinamerika nach dem Ende des Rohstoffbooms.

[CR31] Halperín Donghi T (1994). Geschichte Lateinamerikas von der Unabhängigkeit bis zur Gegenwart.

[CR32] Harvey D (1989). The condition of postmodernity. An enquiry into the origins of cultural change.

[CR33] Held D, McGrew AG, Goldblatt D, Perraton J (1999). Global transformations. Politics, economics, and culture.

[CR34] Hopkins TK, Wallerstein I (1986). Commodity chains in the world-economy prior to 1800. Review.

[CR35] Hung H (2022). Clash of empires. From „Chimerica“ to the „New Cold War“.

[CR36] Ikenberry GJ (2008). The rise of China and the future of the West: Can the liberal system survive?. Foreign Affairs.

[CR37] Kagarlitsky B, Puder J, Schmalz S (2022). „The whole world is becoming more like Russia.“ A conversation on deglobalization in the wake of the war in Ukraine. Berliner Journal für Soziologie.

[CR38] Leggewie C (2003). Die Globalisierung und ihre Gegner.

[CR39] Lessenich S (2016). Neben uns die Sintflut. Die Externalisierungsgesellschaft und ihr Preis.

[CR40] Lobo, S. (2021). Labiler Kapitalismus. Die Globalisierung ist heute anders kaputt als früher. *Spiegel Online *vom 13.10.2021. https://www.spiegel.de/netzwelt/netzpolitik/labiler-kapitalismus-die-globalisierung-ist-heute-anders-kaputt-als-frueher-kolumne-a-1dad7098-eae3-4a3d-a99d-28eaafe44a08. Zugegriffen: Aug. 2022.

[CR41] Löw M, Sayman V, Schwerer J, Wolf H, Löw M, Sayman V, Schwerer J, Wolf H (2021). Am Ende der Globalisierung: Über die Refiguration von Räumen. Re-Figuration von Räumen.

[CR42] Marx K, Engels F, Marx K, Engels F (1972). Manifest der Kommunistischen Partei. Werke.

[CR20212] Mearsheimer, J. (2021). The inevitable rivalry: America, China, and the tragedy of great-power politics. *Foreign Affairs*. 100(6), 48–55.

[CR43] Menzel U (1998). Globalisierung versus Fragmentierung.

[CR44] Menzel U (2021). Corona und die gefesselte Globalisierung. Berliner Journal für Soziologie.

[CR45] Nederveen Pieterse J (2021). Connectivity and global studies.

[CR46] Osterhammel J, Petersson NP (2007). Geschichte der Globalisierung. Dimensionen, Prozesse, Epochen.

[CR47] Pohle J, Voelsen D (2022). Das Netz und die Netze. Vom Wandel des Internets und der globalen digitalen Ordnung. Berliner Journal für Soziologie.

[CR48] Pries L (2002). Transnationalisierung der sozialen Welt?. Berliner Journal für Soziologie.

[CR49] Pries L (2005). Configurations of geographic and societal spaces: A sociological proposal between „methodological nationalism“ and the „spaces of flows“. Global Networks.

[CR50] Quijano A (2000). Coloniality of power and eurocentrism in Latin America. International Sociology.

[CR51] Robinson WI (2004). A theory of global capitalism. Production, class, and state in a transnational world.

[CR52] Rodrik D (2011). Das Globalisierungs-Paradox. Die Demokratie und die Zukunft der Weltwirtschaft.

[CR53] Rosa H, Dörre K, Lessenich S (2017). Appropriation, activation and acceleration: The escalatory logics of capitalist modernity and the crises of dynamic stabilization. Theory, Culture & Society.

[CR54] Sassen S (1991). The global city. New York, London, Tokyo.

[CR55] Sassen S (1999). Globalization and its discontents. Essays on the new mobility of people and money.

[CR56] Scherrer C (2022). Krieg und Abschottung: Das Ende der Globalisierung?. Blätter für Deutsche und Internationale Politik.

[CR57] Schirm SA (2019). In pursuit of self-determination and redistribution: emerging powers and Western anti-establishment voters in international politics. Global Affairs.

[CR58] Schmalz S (2018). Machtverschiebungen im Weltsystem. Der Aufstieg Chinas und die große Krise.

[CR20202] Schmalz, S. (2020). Plädoyer für selektive De-Globalisierung. *Luxemburg – Gesellschaftsanalyse und linke Praxis*. https://zeitschrift-luxemburg.de/artikel/selektive-de-globalisierung/. Zugegriffen: Aug. 2022.

[CR59] Schmalz S, Kraemer K, Münnich S (2021). Widersprüche der Globalisierung: Der Aufstieg Chinas und der Wirtschaftskrieg mit den USA. Ökonomischer Nationalismus. Soziologische Analysen wirtschaftlicher Ordnungen.

[CR60] Schmalz S, Gräf H, Köncke P, Schneidemesser L (2022). Umkämpfte Globalisierung: Amerikanische und europäische Reaktionen auf Chinas Aufstieg im Hochtechnologiebereich. Berliner Journal für Soziologie.

[CR61] Sklair L (2001). The transnational capitalist class.

[CR62] Steger MB (2017). Globalization: A very short introduction.

[CR63] Stolz B, Held M, Georges G, Boulouchos K (2022). Techno-economic analysis of renewable fuels for ships carrying bulk cargo in Europe. Nature Energy.

[CR64] Streeck W (2021). Zwischen Globalismus und Demokratie. Politische Ökonomie im ausgehenden Neoliberalismus.

[CR65] Suder K, Kallmorgen JF (2022). Das geopolitische Risiko. Unternehmen in der neuen Weltordnung.

[CR66] Therborn G (2000). Globalizations: Dimensions, historical waves, regional effects, normative governance. International Sociology.

[CR67] Wallerstein IM (2000). The essential Wallerstein.

[CR68] Wallerstein IM, Wallerstein I, Collins R, Mann M, Derluguian G, Calhoun G (2013). Structural crisis, or why capitalists may no longer find capitalism rewarding. Does capitalism have a future?.

[CR69] Walter S (2021). The backlash against globalization. Annual Review of Political Science.

[CR70] Weiss A (2005). The transnationalization of social inequality: Conceptualizing social positions on a world scale. Current Sociology.

[CR71] Wimmer A, Glick Schiller N (2003). Methodological nationalism, the social sciences, and the study of migration: An essay in historical epistemology. The International Migration Review.

[CR72] Windolf P (2005). Finanzmarkt-Kapitalismus. Analysen zum Wandel von Produktionsregimen.

[CR73] Wolf M (2005). Why globalization works.

[CR74] Zürn M (1998). Regieren jenseits des Nationalstaates. Globalisierung und Denationalisierung als Chance.

